# Ranitidine attenuates myocardial fibrosis by suppressing histamine/H2 receptor signaling and FAK/Src activation

**DOI:** 10.3389/fphar.2026.1874499

**Published:** 2026-07-22

**Authors:** Liwen Yang, Jiexin Zhang, Jun Zhao, Meiling He, Qiaoqi Zheng, Haoxin Chen, Yonghe Hu, Pan Long, Xin Chen

**Affiliations:** 1 Department of Clinical Laboratory Medicine, Affiliated Hospital of Southwest Jiaotong University/The Third People’s Hospital of Chengdu, Chengdu, Sichuan, China; 2 Hubei Key Laboratory of Nanomedicine for Neurodegenerative Diseases, School of Chemistry, Chemical Engineering and Life Science, Wuhan University of Technology, Wuhan, Hubei, China; 3 Hospital of Chengdu University of Traditional Chinese Medicine, Chengdu University of Traditional Chinese Medicine, Chengdu, Sichuan, China; 4 Department of Ophthalmology, The General Hospital of Western Theater Command, Chengdu, Sichuan, China

**Keywords:** FAK/Src, fibroblast, myocardial fibrosis, ranitidine, TAC

## Abstract

**Objective:**

Myocardial fibrosis (MF) is a pathological process often triggered by chronic inflammation and pressure overload. This study aimed to investigate the therapeutic potential of ranitidine, a histamine H2 receptor antagonist, on MF and explore its underlying mechanisms, focusing on the FAK/Src pathway and inflammatory responses.

**Methods:**

A mouse model of MF was established by transverse aortic constriction (TAC). Cardiac function was assessed by echocardiography. Histopathological changes, collagen deposition, mast cell infiltration, and the expression of histamine and its H2 receptor were examined. *In vitro*, NIH/3T3 fibroblasts were stimulated with TGF-β1 to induce fibrotic activation. The effects of ranitidine on collagen synthesis, the expression of fibrotic markers (α-SMA, Collagen I/III), and the phosphorylation of FAK/Src were evaluated.

**Key Findings:**

Ranitidine treatment significantly improved TAC-induced cardiac dysfunction and attenuated myocardial fibrosis, as evidenced by reduced collagen deposition. It also markedly decreased mast cell infiltration and the expression of histamine and H2 receptor in cardiac tissues. In TGF-β1-stimulated fibroblasts, ranitidine dose-dependently reduced collagen synthesis, downregulated α-SMA, Collagen I, and Collagen III expression, and suppressed the activation of the FAK/Src pathway.

**Conclusion:**

Our findings demonstrate that ranitidine ameliorates pressure overload-induced myocardial fibrosis, likely in association with attenuation of histamine/H2 receptor-related inflammatory responses and reduced FAK/Src activation. This study suggests the repurposing potential of ranitidine for treating MF.

## 1 Introduction

Fibrosis-induced organ failure accounts for 45% of deaths in developed nations, making it a major public health issue due to the immense cost burden it imposes on society and people (Rubino et al., 2023). Myocardial fibrosis (MF) is a prevalent pathogenic characteristic of many cardiovascular disorders, including hypertension, myocardial infarction, diabetic cardiomyopathy, and atherosclerosis (López et al., 2015). MF is a pathological condition that results from excessive cardiac fibroblast proliferation, extracellular matrix collagen deposition, and distribution, all of which cause structural heart damage and myocardial dysfunction (López et al., 2015). Even grave consequences including arrhythmia, heart failure, and sudden cardiac death can be brought on by MF (López et al., 2015). The key effector cells in MF and the primary source of matrix proteins are activated fibroblasts and myofibroblasts (Frangogiannis, 2021). However, there are still few medications that may effectively treat the underlying causes of MF, and the need for novel anti-myocardial fibrosis medications is urgent.

It has been known for 50 years that histamine could stimulate collagen synthesis (Cohen et al., 1972). In human PSC, Mdr2^−/−^ mice, and CCA xenograft models, histamine’s interaction with one of the 4 G protein-coupled histamine receptors encourages liver fibrosis (Jones et al., 2016). For patients with persistent heart failure, inhibiting the histamine receptor H2 is particularly beneficial (Kim et al., 2006). Ranitidine, a histamine receptor H2 antagonist used clinically to treat gastric acid hypersecretion, has been found to exert antifibrotic effects potentially by inhibiting the proliferation of fibroblasts with the migration of cholangiocarcinoma cells, with potential therapeutic effects in the treatment of pulmonary fibrosis and bile duct injuries (Jordana et al., 1988; Kennedy et al., 2020). However, the roles of histamine H2 receptor antagonists in myocardial fibrosis are currently unknown. As a result, the identification of novel practical applications for ranitidine and the clarification of its possible mechanism of action for the treatment of myocardial fibrosis offer a theoretical and experimental foundation for the clinical development of novel antifibrotic medicines.

Focal adhesion kinase (FAK) and Src are two essential non-receptor tyrosine kinases that aggregate at the cell-extracellular matrix (ECM) interface and not only provide scaffolding functions, but also stimulate cell motility, cell cycle progression, and participate in cellular signalling (Su et al., 2016). It is becoming apparent that activation of the FAK/Src cascade reaction plays a crucial role in the fibrotic transformation of fibroblasts. Transforming growth factor (TGF)-β1 stimulates FAK/Src activation in dermal fibroblasts, and inhibiting the Src family of kinases reduces hepatic fibrosis (Su et al., 2016; Du et al., 2020). In recent years, FAK/Src activation has been proposed as a potential therapeutic target in fibrotic illnesses to enhance fibroblast migration with myofibroblast transformation (Li and Frangogiannis, 2022).

In this study, we used TAC to create a pressure overload-induced myocardial fibrosis model in mice, and used TGF-β1-stimulated NIH/3T3 fibroblasts as an *in vitro* fibroblast activation model relevant to fibrosis. We then evaluated the effects of ranitidine on myocardial fibrosis and examined the involvement of FAK/Src signaling.

## 2 Materials and methods

### 2.1 Antibodies and reagents

The following primary antibodies were used in this study: anti-HRH2 (Biosynthesis, bs-6664R; 1:400 for immunohistochemical staining), anti-p-Src (Signalway Antibody, 11,091; 1:1000 for western blotting), anti-FAK and anti-p-FAK (Zhengneng Biotechnology, R24276 and R22958; both 1:1000 for western blotting), anti-α-SMA (Proteintech, 14395-1-AP; 1:5000 for western blotting), anti-Collagen I (Proteintech, 14695-1-AP; 1:3000 for western blotting), anti-Collagen III (Proteintech, 14695-1-AP; 1:3000 for western blotting), and anti-Src (Proteintech, 11097-1-AP; 1:1000 for western blotting). Histamine (Sigma, H7403; 1:100 for immunohistochemical staining) and an HRP-conjugated goat anti-rabbit secondary antibody (Servicebio, G1213; 1:5000 for western blotting and 1:200 for immunohistochemical staining) were also used.

Key reagents included ranitidine (Macklin, R838252, purity >98%), valsartan (Macklin, V844023, purity >95%), and TGF-β1 (MedChemExpress, HY-P7117, purity >95%).

### 2.2 Animal care

Specific-pathogen-free (SPF) male C57BL/6J mice (8 weeks old, 20–25 g) were obtained from Chengdu Dashuo Experimental Animal Co., Ltd (China; Animal Qualification Certificate No. SCXK (Chuan) 2020–030). The mice were housed under standard conditions with a 12 h light/dark cycle, ambient temperature of 25 °C ± 2 °C, humidity of 55% ± 5%, adequate ventilation, minimal noise, and free access to food and water. All experimental procedures were approved by the Ethics Committee of the General Hospital of the Western Theatre Command of the Chinese People’s Liberation Army (Approval No.: 2022ky028-1; Lot No.: 2022EC2-ky004) on 14 June 2022.

### 2.3 Animal experiments

A mouse model of pressure overload-induced myocardial fibrosis was established by transverse aortic constriction (TAC), as previously described (Silva et al., 2021). Briefly, mice were anesthetized via intraperitoneal injection of 0.3% pentobarbital sodium and intubated for mechanical ventilation. Ventilator settings were as follows: respiratory rate, 110 breaths/min; inspiration-to-expiration ratio, 1:1; tidal volume, 2 mL. The sternum was clipped to expose the second intercostal space, and the thymus gland was carefully separated using microscopic forceps to reveal the aortic arch. A 7–0 silk suture was used to tie the aorta against a 27-gauge L-type needle placed between the left common carotid artery and the innominate artery. The needle was then promptly removed to induce partial aortic constriction. The sternum and skin were sutured with 5–0 thread, and the surgical site was disinfected with iodophor. After regaining consciousness, mice were housed in individual cages and administered penicillin sodium (5,000 U) for three consecutive days to prevent infection. In the sham group, the thoracic cavity was opened and sutured without aortic ligation. A total of 30 mice were included in this study. Six mice underwent sham surgery, and the remaining 24 mice were subjected to TAC surgery. Two weeks after TAC surgery, 19 of the 24 TA C-operated mice survived, corresponding to a postoperative survival rate of 79%. The postoperative deaths occurred before drug allocation and were considered to be mainly related to the TAC procedure. After TAC surgery, Doppler ultrasonography was performed to assess blood flow at the aortic arch ligation site. Mice showing no significant increase in blood flow velocity compared with the sham group were excluded (Supplementary Figure S1). All 19 surviving TAC mice met the modeling criteria and were included in the subsequent experiments.

Mice were then assigned to four groups: Sham (n = 6), TAC model (n = 7), Val group (valsartan, 10 mg/kg/day, positive control, n = 6), and RN group (ranitidine, 400 mg/kg/day, n = 6). All treatments were administered by oral gavage for 2 weeks. The mouse-equivalent reference dose was estimated using body surface area-normalized interspecies dose conversion. Based on the usual adult clinical dose, the baseline mouse-equivalent dose of ranitidine was approximately 40–50 mg/kg/day. The dose of 400 mg/kg/day was therefore selected as a supra-equivalent pharmacologically active dose for this exploratory mechanistic study, supported by our preliminary efficacy and tolerability observations in the TAC model. During the ranitidine treatment period, no treatment-related mortality, obvious abnormal behavior, or severe body weight loss was observed, indicating that this dose was well tolerated by the treated animals under the present experimental conditions.

### 2.4 Ultrasound imaging

Cardiac function was evaluated by echocardiography using the Vevo 1100 Small Animal Ultrasound Imaging System (Visual Sonics, Canada) equipped with a 30 MHz transducer. Both B-mode and M-mode images were acquired from the parasternal left ventricular long-axis view. Key cardiac parameters, including left ventricular internal diameter at end-systole (LVIDs), ejection fraction (EF%), and fractional shortening (FS%), were measured using the built-in analysis software.

### 2.5 Histopathological analysis

Ventricular tissue samples were collected from mice and fixed in 4% paraformaldehyde for 48 h. Following dehydration, the tissues were embedded in paraffin, sectioned, and baked at 58 °C for 2 h. Histological staining was performed according to the manufacturer’s protocols using the following commercial kits: HE Staining Kit (G1120), Masson Staining Kit (G1340), Sirius Red Staining Kit (G1472), and Toluidine Blue Stain (G3670; all from Solarbio, China).

### 2.6 Immunohistochemical staining

Deparaffinized sections were subjected to antigen retrieval by boiling in 0.01 M citrate buffer (pH 6.0). Endogenous peroxidase activity was then quenched by incubation with 3% hydrogen peroxide. After blocking with 5% goat serum, the sections were incubated sequentially with primary and secondary antibodies, with PBS washes between each step. Color development was performed using DAB, and nuclei were counterstained with hematoxylin. Following differentiation in hydrochloric acid alcohol, sections were washed in tap water, dehydrated, cleared, and mounted with neutral resin for microscopic examination.

### 2.7 Cell culture and treatments

The mouse embryonic fibroblast cell line NIH/3T3 was used as a reproducible *in vitro* fibroblast activation model rather than as a cardiac-specific fibroblast model. NIH/3T3 cells were maintained in high-glucose DMEM supplemented with 10% fetal bovine serum and 1% penicillin/streptomycin. When cells reached 60%–70% confluence, they were serum-starved for 12 h. Subsequently, the control group was cultured in serum-free medium, while the model group was treated with 15 ng/mL TGF-β1 in serum-free medium for 48 h. In the treatment groups, cells were stimulated with 15 ng/mL TGF-β1 and concurrently exposed to ranitidine at concentrations of 0.1 μM, 1 μM, or 10 μM. After 48 h of treatment, cells were harvested for subsequent analysis.

### 2.8 Estimation of cellular total collagen

Cells were seeded into 12-well plates and harvested upon reaching 60%–70% confluence. Total collagen content was measured using the Sirius Red Total Collagen Assay Kit (Chondrex, #9062) according to the manufacturer’s instructions.

### 2.9 Quantitative real-time PCR

Total RNA was extracted from cells using Trizol reagent, and its concentration was determined. cDNA was synthesized following the instructions of the reverse transcription kit (Yisheng Bio-technology Co., China, #11142). Quantitative real-time PCR was performed using SYBR GREEN I (Tsingke Biotechnology Co., Ltd., China, #TSE201) with GAPDH as the internal control to assess the mRNA expression levels of Collagen I, Collagen III, and α-SMA. The primer sequences used are listed in Table 1. The PCR protocol consisted of an initial denaturation at 95 °C for 2 min, followed by 40 cycles of 95 °C for 15 s and 58 °C for 30 s. A melting curve analysis was conducted from 60 °C to 95 °C to verify amplification specificity. Relative gene expression was calculated using the 2^–ΔΔCt^ method.

**TABLE 1:** Sequences of the primers.

Name	Primer sequence
Collagen I	Forward:5′-GCCTAGCAACATGCCAATATTT-3′
	Reverse: 5′-GAA​TAC​TGA​GCA​GCA​AAG​TTC​C-3′
Collagen III	Forward: 5′-GCG​AGC​GGC​TGA​GTT​TTA​TG-3′
	Reverse: 5′-GCA​GCT​CAG​AGT​AGC​ACC​AT-3′
α-SMA	Forward: 5′-CCC​TGA​AGA​GCA​TCC​GAC​A-3′
	Reverse: 5′-CCA​GAG​TCC​AGC​ACA​ATA​CCA-3′
GAPDH	Forward: 5′-AGG​TCG​GTG​TGA​ACG​GAT​TTG-3′
	Reverse: 5′-GGG​GTC​GTT​GAT​GGC​AAC​A-3′

### 2.10 Western blotting

Cells were lysed using RIPA buffer, and protein concentrations were determined with a BCA assay kit (Beyotime Biotechnology, China, P0012). After loading equal amounts of protein, samples were separated by SDS-PAGE at 120 V and transferred onto a PVDF membrane by wet transfer at 300 mA for 70 min. The membrane was blocked with 5% BSA at room temperature for 90 min and then incubated overnight with the primary antibody at 4 °C. After washing with TBST, the membrane was incubated with an HRP-conjugated secondary antibody at room temperature for 60 min. When sequential probing was required, membranes were stripped using stripping buffer according to the manufacturer’s instructions, washed, re-blocked, and incubated with the next primary antibody. Protein bands were visualized using an ECL substrate, and band intensities were quantified with a gel imaging system.

### 2.11 Statistics

Data are presented as mean ± standard deviation (SD). All statistical analyses were performed using GraphPad Prism software (version 8.0). Comparisons among multiple groups were performed using one-way analysis of variance (ANOVA) followed by Tukey’s multiple comparison test; Bonferroni correction was applied where appropriate for planned multiple pairwise comparisons. For comparisons between two groups, an unpaired two-tailed Student’s t-test was used when appropriate. A p-value of less than 0.05 was considered statistically significant.

## 3 Results

### 3.1 Ranitidine improves TAC-induced cardiac dysfunction in mice

**FIGURE 1:**
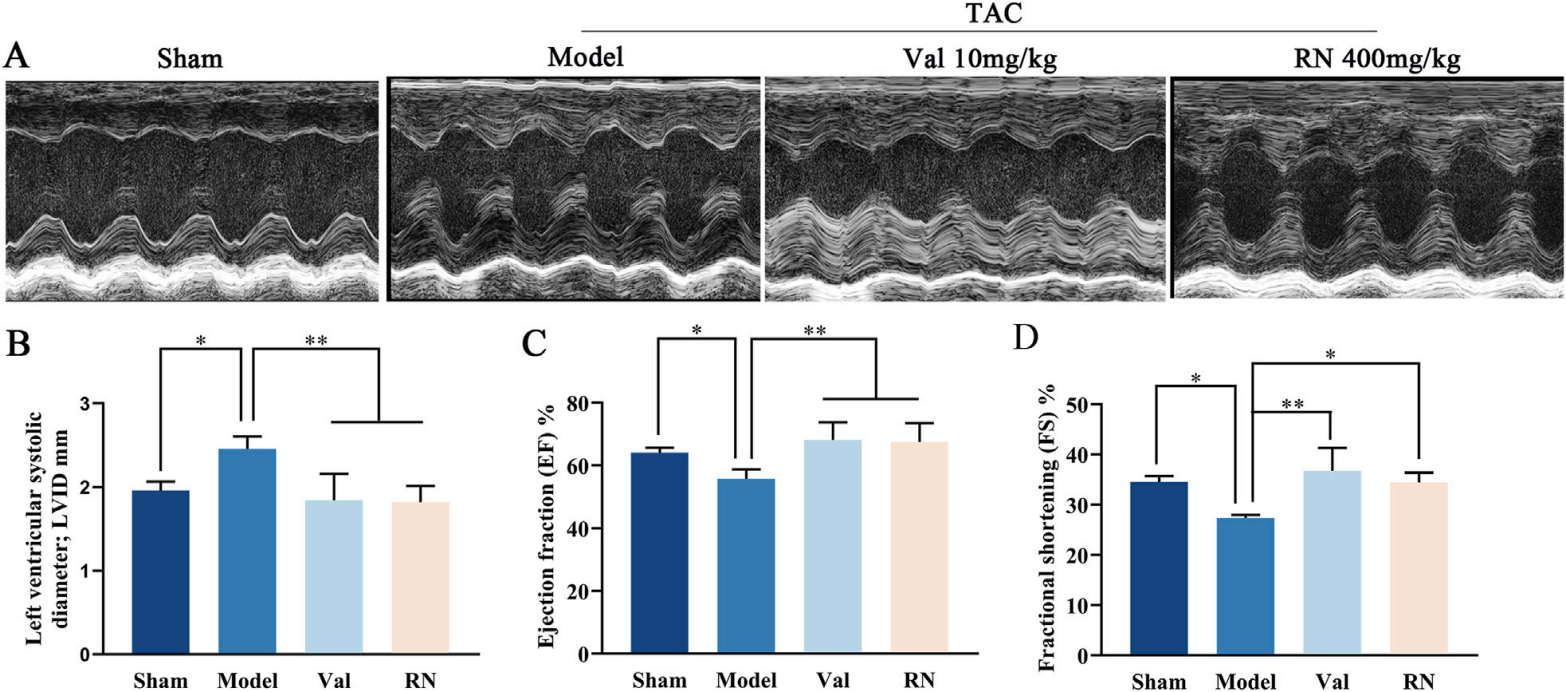
Ranitidine ameliorates TAC-induced cardiac dysfunction in mice. **(A)** Representative M-mode echocardiograms. **(B)** Left ventricular internal diameter at end-systole (LVIDs, mm). **(C)** Ejection fraction (EF%). **(D)** Fractional shortening (FS%). Data are presented as mean ± SD. *p < 0.05, **p < 0.01. Sham, n = 6; TAC, n = 6; Val, n = 6; RN, n = 6.

To evaluate the effect of ranitidine on pressure overload-induced cardiac remodeling, cardiac function and morphology were assessed by echocardiography 4 weeks after TAC surgery (Figure 1). Compared with sham-operated mice (LVIDs: 1.960 ± 0.11 mm; EF%: 64.07% ± 1.62%; FS%: 34.57% ± 1.15%), TAC-induced model mice exhibited a significant increase in LVIDs (2.457 ± 0.15 mm, p < 0.05) and marked decreases in EF% (55.81% ± 2.95%) and FS% (27.34% ± 0.63%) (all p < 0.05), indicating impaired left ventricular systolic function. Ranitidine treatment effectively reversed these changes, restoring LVIDs to 1.820 ± 0.19 mm (p < 0.01 vs. model), EF% to 67.48% ± 6.11% (p < 0.01), and FS% to 34.44% ± 1.98% (p < 0.05). These results demonstrate that ranitidine ameliorates TAC-induced cardiac systolic dysfunction in mice.

### 3.2 Ranitidine attenuates TAC-induced myocardial fibrosis in mice

**FIGURE 2:**
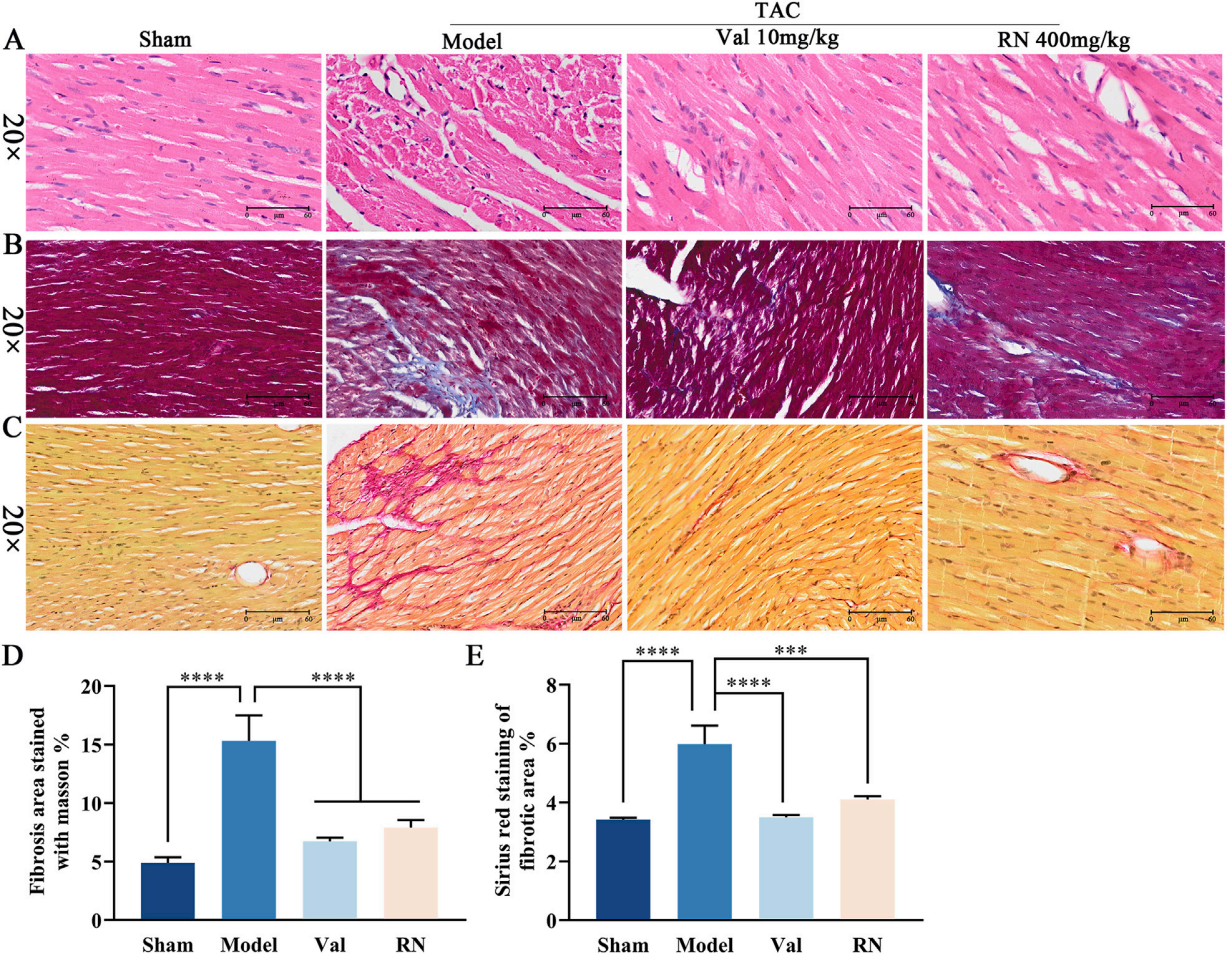
Ranitidine attenuates TAC-induced myocardial histopathological changes and collagen deposition in mice. **(A)** Representative HE staining. **(B)** Representative Masson’s trichrome staining. **(C)** Representative Sirius red staining. **(D)** Quantification of fibrotic area based on Masson’s trichrome staining. **(E)** Quantification of fibrotic area based on Sirius red staining. Data are presented as mean ± SD. ***p < 0.001, ****p < 0.0001. Sham, n = 6; TAC, n = 6; Val, n = 6; RN, n = 6.

Histological analysis by HE staining (Figure 2A) revealed structurally intact cardiomyocytes and well-aligned myocardial fibers in sham-operated mice. In contrast, the TAC group exhibited disorganized myocardial architecture, disrupted fibers, local connective tissue proliferation, and scattered inflammatory cell infiltration on descriptive histological assessment. Ranitidine treatment ameliorated these pathological changes, leading to more regular cell morphology, reduced intercellular space, and less apparent inflammatory infiltration.

Masson and Sirius red staining (Figures 2B–E) further confirmed extensive collagen deposition in TAC mice, manifested as blue (Masson) or red (Sirius red) fibrillar staining in the interstitial areas, along with widespread fibrotic replacement of normal myocardium. Ranitidine treatment significantly reduced collagen fiber accumulation and decreased the fibrotic area compared with the TAC group (p < 0.001), indicating attenuation of myocardial fibrosis.

### 3.3 Ranitidine reduces myocardial histamine enrichment and H2 receptor expression

**FIGURE 3:**
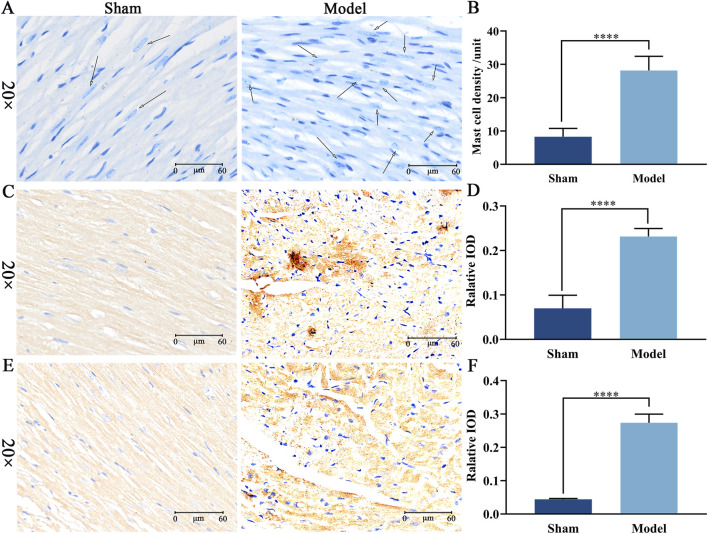
Ranitidine reduces cardiac mast cell infiltration and suppresses histamine/H2 receptor expression in TAC-induced mice. **(A)** Representative toluidine blue staining of mast cells. Magnification ×20, Scale bar = 60 μm. **(B)** Quantification of mast cell density. **(C)** Representative immunohistochemical staining for histamine. **(D)** Quantification of histamine-positive area. **(E)** Representative immunohistochemical staining for histamine H2 receptor. **(F)** Quantification of H2 receptor-positive area. Data are presented as mean ± SD. ****p < 0.0001. Sham, n = 6; TAC, n = 6; Val, n = 6; RN, n = 6.

Toluidine blue staining (Figures 3A,B) revealed a significant increase in mast cell infiltration in the hearts of TAC-induced mice, which was markedly attenuated by ranitidine treatment. Immunohistochemical analysis (Figures 3C–F) further demonstrated stronger positive staining for both histamine and the histamine H2 receptor in myocardial tissues of TAC mice compared with sham controls. Ranitidine intervention substantially reduced the expression of both molecules, suggesting inhibition of histamine/H2 receptor-related signaling under pressure overload.

### 3.4 Ranitidine attenuates TGF-β1-induced collagen accumulation in fibroblasts

**FIGURE 4:**
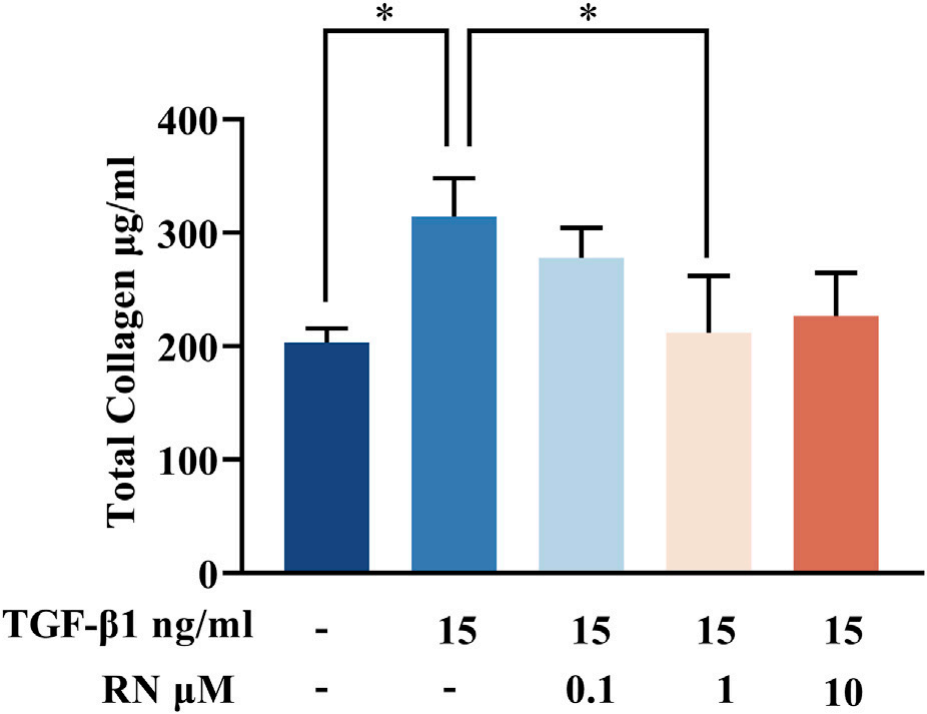
Ranitidine inhibits TGF-β1-induced total collagen accumulation in NIH/3T3 fibroblasts. Total collagen content was measured by Sirius red assay. Data are presented as mean ± SD from three independent experiments. *p < 0.05.

To evaluate the anti-fibrotic effect of ranitidine *in vitro*, NIH/3T3 fibroblasts were stimulated with 15 ng/mL TGF-β1 to establish a fibroblast activation model relevant to fibrosis. Total collagen content was measured using the Sirius red assay. As shown in Figure 4, TGF-β1 stimulation significantly increased intracellular collagen levels compared with the control group (p < 0.05). Treatment with 1 μM ranitidine markedly attenuated this increase, indicating that ranitidine suppresses collagen accumulation in fibroblasts.

### 3.5 Ranitidine attenuates TGF-β1-induced fibrotic protein expression and FAK/Src activation

**FIGURE 5:**
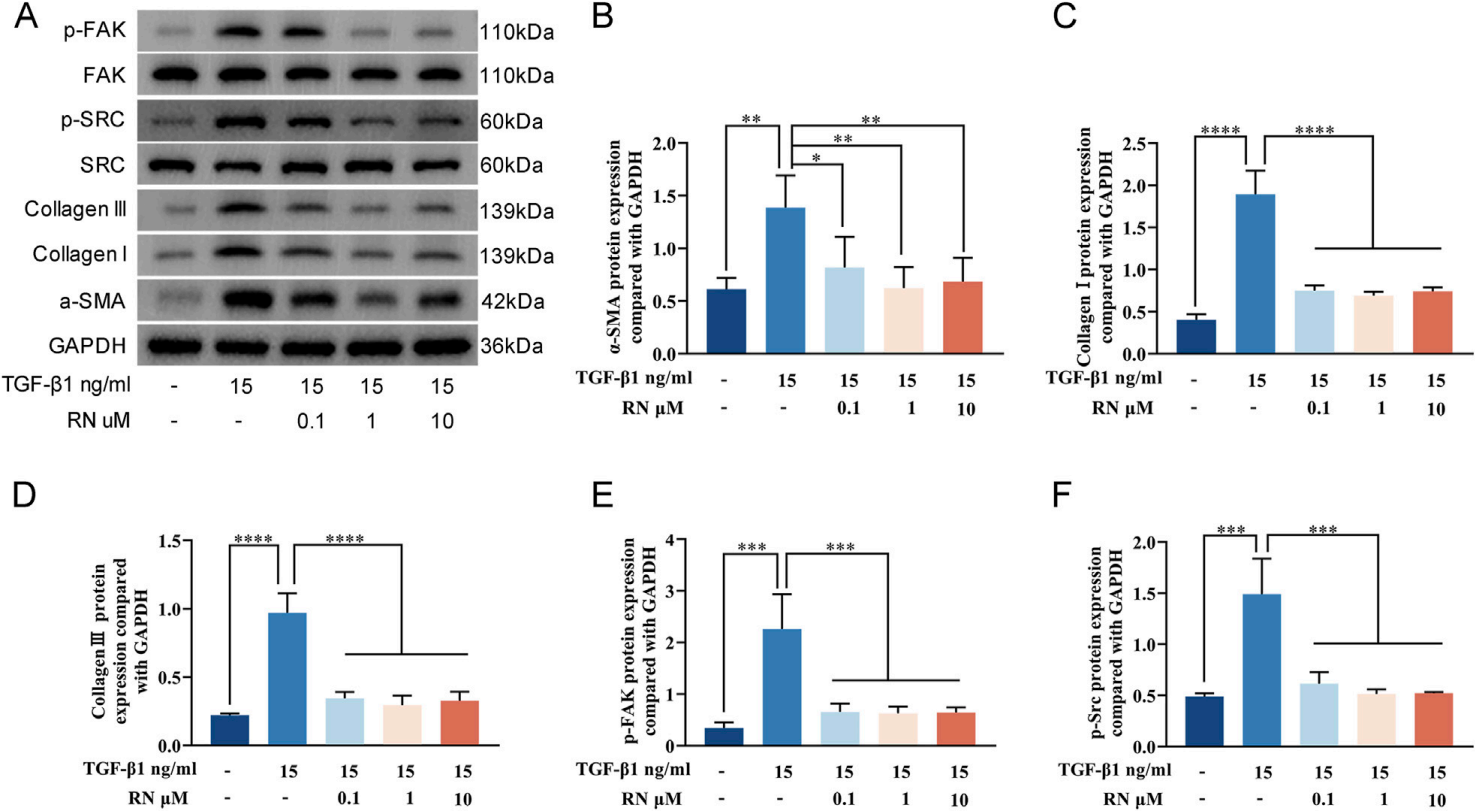
Ranitidine inhibits the expression of fibrosis-related proteins and reduces FAK/Src activation in TGF-β1-stimulated fibroblasts. **(A)** Representative Western blot images of p-FAK, FAK, p-Src, Src, α-SMA, Collagen I, Collagen III, and GAPDH in fibroblasts treated with TGF-β1 (15 ng/mL) with or without ranitidine (RN, 0.1, 1, and 10 μM). **(B–D)** Quantification of α-SMA, Collagen I, and Collagen III protein expression. **(E,F)** Quantification of p-FAK and p-Src protein expression. Data are presented as mean ± SD from three independent experiments. *p < 0.05, **p < 0.01, ***p < 0.001, ****p < 0.0001.

α-SMA is a well-established marker of fibroblast activation and myofibroblast differentiation, whereas Collagen I and Collagen III are major extracellular matrix components associated with cardiac fibrosis. As shown in Figures 5A–F, TGF-β1 stimulation markedly increased the protein expression of α-SMA, Collagen I, and Collagen III compared with the control group. In parallel, TGF-β1 significantly enhanced the phosphorylation of FAK and Src, indicating activation of FAK/Src signaling. Ranitidine treatment dose-dependently attenuated the TGF-β1-induced upregulation of these fibrotic proteins and reduced FAK/Src phosphorylation, suggesting that ranitidine-mediated suppression of fibroblast activation is associated with decreased FAK/Src activation.

### 3.6 Ranitidine suppresses TGF-β1-induced fibrotic gene expression

**FIGURE 6:**
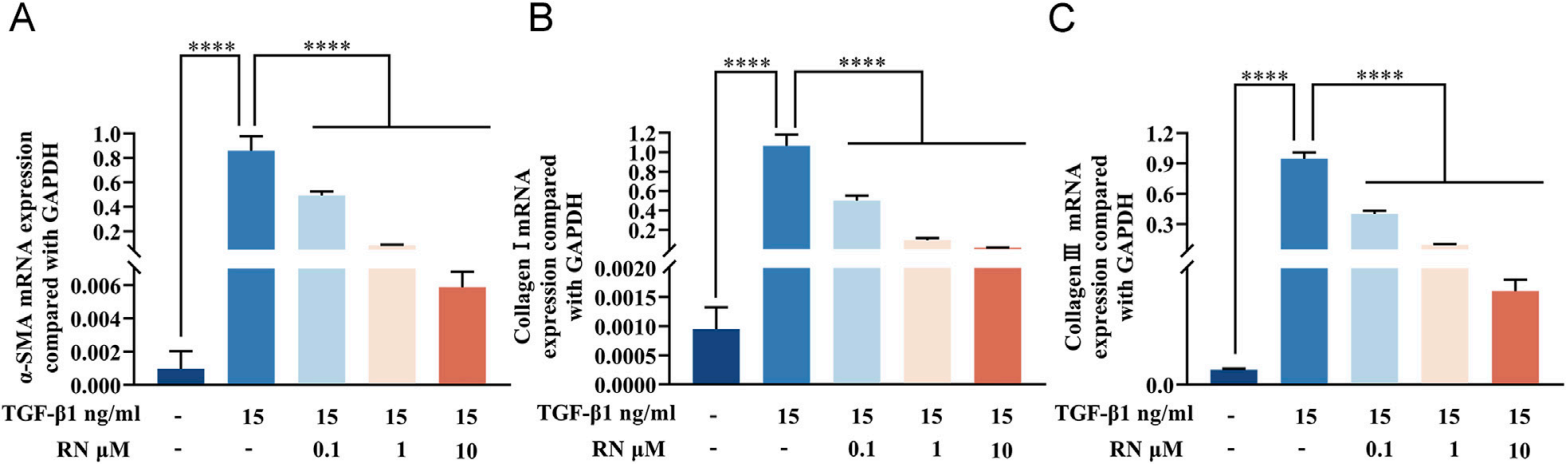
Ranitidine reduces the mRNA expression of fibrosis-related markers in TGF-β1-stimulated fibroblasts. **(A–C)** Relative mRNA expression levels of α-SMA, Collagen I, and Collagen III in fibroblasts treated with TGF-β1 (15 ng/mL) with or without ranitidine (RN, 0.1, 1, and 10 μM). Data are presented as mean ± SD from three independent experiments. ***p < 0.001.

As shown in Figures 6A–C, RT-qPCR analysis revealed that TGF-β1 significantly elevated the mRNA expression levels of α-SMA, Collagen I, and Collagen III relative to the control group. Ranitidine treatment significantly reversed these changes and reduced the transcriptional expression of these fibrotic markers in a dose-dependent manner. These findings indicate that ranitidine suppresses TGF-β1-induced fibrotic gene expression in fibroblasts.

## 4 Discussion

The heart’s structural integrity relies on a collagen-rich extracellular matrix that interconnects cardiomyocytes, fibroblasts, and vascular cells (Gyöngyösi et al., 2017). Under physiological conditions, fibroblasts maintain ECM homeostasis; however, they also serve as the principal effector cells in myocardial fibrosis (Liu et al., 2021). Given the limited regenerative capacity of the adult mammalian heart, tissue repair often occurs through scar formation (Frangogiannis, 2021). Upon injury, fibroblasts activate and differentiate into myofibroblasts, which drive pathological cardiac remodeling by excessive proliferation and ECM deposition (Liu et al., 2021). Persistent myofibroblast activity disrupts collagen turnover, elevating synthesis while impairing degradation (Kong et al., 2014). This leads to the replacement of contractile myocardium by non-contractile fibrotic tissue, resulting in structural and functional impairment that progresses to heart failure. The transition to myofibroblasts is marked by α-smooth muscle actin (α-SMA) expression and increased secretion of collagens, particularly types I and III, which assemble into protofibrils and undergo cross-linking to form mature fibers (Kong et al., 2014; Gyöngyösi et al., 2017).

Transforming growth factor-beta (TGF-β) is a central mediator of fibrotic signaling. Genetic studies have established that TGF-β1 activation promotes cardiac fibrosis, and its myocardial overexpression is sufficient to initiate the process (Frangogiannis, 2021). Based on this, we modeled pressure overload-induced myocardial fibrosis in mice via TAC and assessed fibrotic activation using α-SMA, collagen I, and collagen III as key markers. In parallel, an *in vitro* model was established by treating mouse fibroblasts with TGF-β1. Histological staining revealed substantial collagen deposition and connective tissue hyperplasia in TAC mice, while echocardiography confirmed impaired left ventricular systolic function. Consistently, TGF-β1-stimulated fibroblasts showed elevated mRNA and protein levels of collagen I and III. Together, these pathological, functional, and molecular results validate the successful establishment of the fibrosis models.

Focal adhesion kinase (FAK) and Src often function as a complex that regulates cell proliferation, migration, and survival (Fan et al., 2015). During tissue repair, fibroblasts migrate into injured areas and remodel the ECM via focal adhesions-specialized structures that mediate cell-matrix attachment (Hinz and Gabbiani, 2003). FAK localizes to these sites and facilitates fibroblast-ECM adhesion, with sustained adhesion signaling being a hallmark of activated fibroblasts (Shi-Wen et al., 2009). Src, the first identified proto-oncogene, is regulated by tyrosine phosphorylation and is activated at wound margins in both murine corneal injury and zebrafish models, contributing to all stages of healing (Su et al., 2016). The FAK/Src complex initiates multiple signaling cascades and integrates cellular responses to the ECM. Previous work has implicated FAK/Src in fibrotic diseases across organs, and its inhibition has been reported to attenuate fibrotic progression (Fan et al., 2015). In the present study, ranitidine treatment was associated with reduced FAK/Src phosphorylation in TGF-β1-stimulated fibroblasts, accompanied by decreased fibrotic marker expression and collagen production. These findings support an association between ranitidine treatment and reduced FAK/Src activation, but they do not establish FAK/Src as the direct molecular target of ranitidine.

Histamine, a well-known inflammatory mediator, stimulates fibroblast proliferation and collagen synthesis *in vitro* (Garbuzenko et al., 2002). Its effects are mediated through specific receptors—H1, H2, and H3—all expressed in the heart. Similar to β-adrenergic receptors, H2 receptors couple to stimulatory G proteins in cardiac tissue (Leary et al., 2016). A prospective cohort study linked H2 receptor antagonist use to a lower incidence of heart failure, and H2 receptor knockout mice exhibited preserved cardiac function and reduced fibrosis after aortic banding (Zeng et al., 2014). Ranitidine, a selective H2 receptor antagonist, is widely used to treat peptic ulcer and gastric hypersecretion (Strum, 1983). It demonstrates a favorable safety profile in clinical settings, with infrequent and mild adverse effects (Strum, 1983). Preclinically, ranitidine is 2- to 10-fold more potent than cimetidine in blocking H2-mediated responses and reduces histamine levels in guinea pig atria (Strum, 1983). Ranitidine also successfully reversed hepatic injury, bile duct response, hepatic fibrosis, and inflammation in Mdr2^−/−^ mice (Jones et al., 2016).

In the present study, TAC induced myocardial histamine accumulation in mice, while ranitidine treatment improved systolic function, downregulated histamine and H2 receptor expression, and reduced collagen deposition-effects comparable to the clinical standard valsartan. Several limitations should be acknowledged. First, only a single ranitidine dose was used in the *in vivo* experiment. Although the selected dose was based on body surface area-normalized interspecies dose conversion, preliminary efficacy observations, and tolerability assessment, it was relatively high compared with the baseline mouse-equivalent dose calculated from clinical exposure. Therefore, possible off-target effects cannot be fully excluded. In addition, the current design does not allow characterization of the complete dose-response relationship. Future studies should include low-, medium-, and high-dose ranitidine groups, for example, doses around the baseline equivalent range and higher exposure levels, to determine the minimum effective dose, optimal therapeutic dose, and safety window. Second, inflammatory cell infiltration was evaluated descriptively based on HE staining, and quantitative inflammatory scoring or immune cell-specific staining, such as CD68 for macrophages or Ly6G for neutrophils, was not included in the present study. In our attempt to perform additional immunostaining, the archived unstained sections showed unstable staining signals, likely related to prolonged section storage. Therefore, the present data do not define the inflammatory cell subtypes involved in TAC-induced myocardial remodeling. Future studies using freshly prepared sections and cell-specific markers are needed to further clarify the inflammatory response associated with ranitidine treatment. Third, the present data indicate that ranitidine treatment was associated with reduced histamine/H2 receptor expression and decreased FAK/Src phosphorylation. However, this study does not establish whether ranitidine directly regulates FAK/Src through H2 receptor-dependent signaling or indirectly affects these pathways by modulating mast cell activation or other inflammatory processes. Functional rescue experiments using H2 receptor agonists such as amthamine, comparison with other H2 receptor antagonists such as cimetidine, and pathway-intervention experiments are needed to define the causal relationship.

**FIGURE 7:**
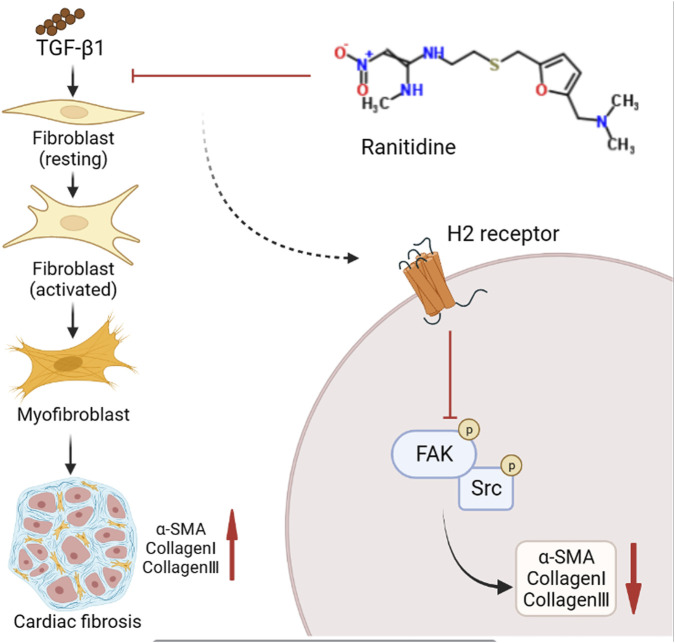
Schematic diagram illustrating the proposed mechanism by which ranitidine alleviates myocardial fibrosis. Ranitidine antagonizes H2 receptor-mediated signaling and is associated with reduced FAK/Src activation. ERK and AKT are shown as representative downstream effectors of FAK/Src based on reported signaling relationships; these downstream components were not directly examined in the present study.

In TGF-β1-stimulated NIH/3T3 cells, ranitidine dose-dependently reduced FAK and Src phosphorylation, downregulated α-SMA, Collagen I, and Collagen III expression, and reduced total collagen synthesis, further supporting its anti-fibrotic effect *in vitro*. NIH/3T3 cells were used in this study as a well-established and reproducible fibroblast model to investigate TGF-β1-induced fibrotic responses. Their advantages include stable growth characteristics and suitability for evaluating fibroblast activation, collagen production, and fibrosis-related signaling pathways under controlled conditions. However, NIH/3T3 cells are embryonic mouse fibroblasts and are not cardiac-specific. Therefore, the *in vitro* findings provide supportive mechanistic evidence but may not fully reflect the biological characteristics of native cardiac fibroblasts. Further studies using primary cardiac fibroblasts would help confirm the cardiac-specific relevance of these findings. The proposed mechanism underlying the anti-fibrotic effects of ranitidine in myocardial fibrosis is illustrated in Figure 7. In summary, our findings support the potential repurposing of ranitidine as an anti-fibrotic agent and indicate that reduced FAK/Src activation may be associated with its anti-fibrotic activity. Future studies should assess the therapeutic effects of ranitidine in more clinically relevant models and clarify the pathway-specific mechanisms underlying its effects.

## 5 Conclusion

In summary, this study demonstrates that ranitidine alleviates pressure overload-induced myocardial fibrosis in mice and inhibits TGF-β1-driven fibrotic activation in fibroblasts. Mechanistically, these effects are associated with suppression of FAK/Src activation, although direct pathway-specific validation is still required. These insights provide a mechanistic foundation for the potential clinical repurposing of ranitidine in the management of cardiac fibrosis.

## Data Availability

The original contributions presented in this study are included in the article and Supplementary Material. Further inquiries regarding the datasets generated during the current study can be directed to the corresponding authors.
