# Supplementary material for: Ranitidine attenuates myocardial fibrosis by suppressing histamine/H2 receptor signaling and FAK/Src activation
Source: Front Pharmacol. 2026 Jul 22;17:1874499. doi: 10.3389/fphar.2026.1874499 (PMC13437269; doi:10.3389/fphar.2026.1874499)
Supplement: Supplementary file 1 [file Table1.docx]

**Supplementary Figure S1. Uncropped Original Western Blot Membrane Images for Figure 5**

Red arrows and circles indicate the target bands, and the expected molecular weights are shown in the labels. The images are original uncropped membrane photographs; annotations were added only to identify the target bands and molecular weights requested during peer review.

**Target proteins and expected molecular weights:**

| **Protein** | **Expected MW** | **Protein** | **Expected MW** |
| --- | --- | --- | --- |
| p-FAK | 110 kDa | FAK | 110 kDa |
| p-Src | 60 kDa | Src | 60 kDa |
| Collagen I | 139 kDa | Collagen III | 139 kDa |
| α-SMA | 42 kDa | GAPDH | 36 kDa |

Lane information: the lanes correspond to the treatment groups shown in Figure 5: control, TGF-β1, and TGF-β1 plus ranitidine (RN, 0.1, 1, and 10 μM).

**Supplementary Figure S1A. α-SMA uncropped membrane image(s). Target band: α-SMA, 42 kDa.**

Red labels identify the target band and expected molecular weight in the original membrane image(s).

**Supplementary Figure S1B. Collagen I uncropped membrane image(s). Target band: Collagen I, 139 kDa.**

Red labels identify the target band and expected molecular weight in the original membrane image(s).

**Supplementary Figure S1C. Collagen III uncropped membrane image(s). Target band: Collagen III, 139 kDa.**

Red labels identify the target band and expected molecular weight in the original membrane image(s).

**Supplementary Figure S1D. FAK uncropped membrane image(s). Target band: FAK, 110 kDa.**

Red labels identify the target band and expected molecular weight in the original membrane image(s).

**Supplementary Figure S1E. p-FAK uncropped membrane image(s). Target band: p-FAK, 110 kDa.**

Red labels identify the target band and expected molecular weight in the original membrane image(s).

**Supplementary Figure S1F. Src uncropped membrane image(s). Target band: Src, 60 kDa.**

Red labels identify the target band and expected molecular weight in the original membrane image(s).

**Supplementary Figure S1G. p-Src uncropped membrane image(s). Target band: p-Src, 60 kDa.**

Red labels identify the target band and expected molecular weight in the original membrane image(s).

**Supplementary Figure S1H. GAPDH uncropped membrane image(s). Target band: GAPDH, 36 kDa.**

Red labels identify the target band and expected molecular weight in the original membrane image(s).
